# Suicide in Inmates in Nazis and Soviet Concentration Camps: Historical Overview and Critique

**DOI:** 10.3389/fpsyt.2016.00088

**Published:** 2016-05-26

**Authors:** Francisco López-Muñoz, Esther Cuerda-Galindo

**Affiliations:** ^1^Faculty of Health Sciences, Camilo José Cela University, Madrid, Spain; ^2^Neuropsychopharmacology Unit, Hospital 12 de Octubre Research Institute (i+12), Madrid, Spain; ^3^Area of Human Anatomy and Embryology, Rey Juan Carlos University, Alcorcón, Madrid, Spain

**Keywords:** suicide, concentration camps, Nazi, Soviets, history of psychiatry

## Abstract

Living conditions in concentration camps were harsh and often inhumane, leading many prisoners to commit suicide. We have reviewed this topic in Nazi concentration camps (KL), Soviet special camps, and gulags, providing some preliminary data for our research. Data show that the incidence of suicide in Nazi KL could be up to 30 times higher than the general population and was also much higher than in Soviet special camps (maybe due to more favorable conditions for prisoners and the abolishment of death penalty), while available data on Soviet gulags are contradictory. However, data interpretation is very controversial, because, for example, the Nazi KL authorities used to cover-up the murder victims as suicides. Most of the suicides were committed in the first years of imprisonment, and the method of suicide most commonly used was hanging, although other methods included cutting blood vessels, poisoning, contact with electrified wire, or starvation. It is possible to differentiate two behaviors when committing suicide; impulsive behavior (contact with electrified barbed wire fences) or premeditated suicide (hanging up or through poison). In Soviet special camps, possible motives for suicides could include feelings of guilt for crimes committed, fear of punishment, and a misguided understanding of honor on the eve of criminal trials. Self-destructive behaviors, such as self-mutilation in gulag camps or prisoners who let themselves die, have been widely reported. Committing suicide in concentration camps was a common practice, although precise data may be impossible to obtain.

## Introduction

Suicides under extraordinary or extreme conditions, such as prisons, war conflicts, or concentration camps, have been studied previously (1–6). Specific studies, including imprisonment (7, 8), deportation (9), exclusion, and torture (10, 11), show a higher rate of suicide in these groups. Suicide in ghettos or transit camps before extermination, as Theresienstadt (12, 13), Nazi concentration camps (*Konzentrationslager*; KL) (14, 15), and Soviet gulags (16) has also been studied.

Prisoners are generally more likely to commit suicide than other people. Suicide rates in prisoners are estimated to be 55–107/100,000 (3, 17, 18). Rates of suicides in prisons in Austria and Switzerland are reported to be between 1.4 and 14 times higher than in the general population (19). More detailed records for Germany between 2003 and 2010 suggest that suicide rates for men serving a prison sentence are about three times higher. (20).

In prewar Berlin, it has been pointed out that suicides were significantly more common in Jewish citizens than in the general population, and timing was often closely associated with anti-semitic persecution (21, 22). Comprehensive data are not available, but in 1942, those who were persecuted after being classified as Jewish according to Nazi race laws were 26 times more likely to commit suicide (rate: 1,480/100,000) than the non-Jewish. Suicides were highly correlated with deportation from Berlin to ghettos and camps in Eastern Europe (23).

Suicidality has been described in Lodtz ghetto: some authors calculated a suicide rate of 85 per 100,000 in 1942 (24). The methods of suicide were recorded as follows: suicide by jumping represented 38%, poisoning 27%, and hanging 17% (21). Other authors reported just a few cases per thousand per year. They explained the relatively low number of suicides by the exhaustion and apathy of inhabitants or because of the strong will to survive, as well as a strong desire to resist the occupiers (13).

The topic of suicides in the Nazi KL has been studied more widely in memoirs than in medical or historical literature (25–27), but those studies based on original documents are scarce. These studies report that the incidence of suicide in Nazi KL was 10–30 times higher than for the general public (28) and was also much higher than in the Soviet special camps, possibly because the prisoners’ living conditions were also much harder (slave labor, medical experiments, etc.), while available data on the Soviet gulags are scarce and contradictory (16, 29).

## Nazi Concentration Camps

The main purpose of Nazi KL existence was to eliminate the Nazi Government’s enemies. In Nazi KL history, two periods must be differentiated, the prewar period from 1933 to 1939 and the war period from October 1939 to the end of war in 1945 (15). During the first phase, before the outbreak of the war, legal officials investigated dubious cases of death in the KL (most of all, in Dachau), including alleged suicides, but the SS (*Schutzstaffel*) authorities covered up the murder victims as suicides (21). Nazi leaders considered that the judiciary was inadequate for the implementation of the Third Reich’s racial and political agenda (30, 31). In the second phase, SS courts were in charge of investigating all deaths of camp inmates, including suicides, with complete independence from the judiciary (32, 33). The central Inspection of Concentration Camps (IKL) administered all KL in Germany and was located at Oranienburg from 1938. IKL controlled imprisoner’s life conditions, forced labor, punishment, and medical experiments (34, 35).

### Epidemiologic Data

Some authors have reported suicides in Nazi KL based on psychiatric interviews with the former prisoners. They described suicide as more frequent in those inmates who suffered the cruelest abuse, suffering from infectious diseases, forced to participate in medical experiments, during periods of mass extermination, and generally in autumn and winter (36). Some authors argue that suicides were extremely high in Nazi KL based on witness testimonies (2, 15, 37–40). In contrast, others suggest that suicides were not so common and argue that in survivor’s memories, under extreme conditions, exists an increase in the self-preservation instinct (27, 41–43). Other authors have estimated that suicides amounted to 25,000–100,000 per year based on testimonies (38). Compared to actual national suicide rates (60 per 100,000 per year), these rates are significantly high (24). Our group, in a preliminary report, has identified 222 cases of suicide in Sachsenhausen KL (44). But no precise data exist from which the suicide rate in KL can be calculated.

There are several problems that make difficult a correct approach to this analysis (a) in the Nazi KL, mostly after 1940, suicides frequently passed unnoticed because death was so common, and only suicides committed by a well-known inmate or by a terrible method were noticed (27); (b) suicide ratios may vary significantly depending on the period studied; suicide levels must have been raised because the camp populations increased in 1937–1938, with the numerous criminals and Jews imprisonment. Baganz (45) suggests that suicide levels in Sachsenhausen camp rose from 7 per month in 1937 to 33 per month in 1938; (c) the SS covered up the murder victims as suicides, which make the counting and interpretation of such suicides very problematic (15). In some cases of famous inmates, they preferred to cover-up the murder to avoid one scandal; and (d) finally, in most cases, data are incomplete mainly because Nazis destroyed documents when leaving the camps at the end of the war.

### Profile of Suicidal Inmate

In Nazi KL, men and women of different age, race, nationality, profession, and social strata committed suicide. Some authors assess that suicides were most often committed by Jewish prisoners due to the fact that they were the largest group. But the Jewish group was extraordinarily inhomogeneous, composed of individuals from various social strata, cultures, and language groups (46). There are frequent reports of suicides committed by Jewish population in Germany, mostly after the Nazi Party came to power and Nuremberg rules were approved (47–49). In other smaller groups, such as communists or Jehovah’s, witnesses’ cohesion was higher (50) and maybe the suicide rate was lower, but it depends on KL and year. Our preliminary report confirms a higher rate of suicides in Sachsenhausen KL among Protestant and Catholic population than in Jews (44). Suicides were overwhelmingly committed by male, reflecting the fact that the majority of KL prisoners were men.

### Moment of Suicidal Act

Inmates, especially in their first period of imprisonment, are often desperate about their lack of freedom and the strict rules (51, 52). In Nazi time, suicides committed during transportation to the KL are reported (53). Oral testimonies report that the majority of suicides were committed in the first years of camp existence (22, 54). Maybe, this fact is related to the special repression during the first years. Political prisoners are reported to commit suicide in order to avoid betraying bearers of secrets under torture (15, 55), and in the first years of existence of camps they committed suicide encouraged by SS authorities (39). In large KL (such as Auschwitz), it has been described that Jewish prisoners frequently committed suicide when they were selected for the *Sonderkommandos* (task force) or for extermination (42).

### Manners of Suicide

The methods to commit suicide in Nazi KL were varied, although these methods are related to the internal structure of the camps. For example, the camp authorities confiscated all knives and razor blades to avoid committing suicides by cutting blood vessels (56). For Theresienstadt ghetto, data from archival sources are available: until 1943, out of 430 people, 285 people had committed suicide by poisoning (barbital, Veronal^®^), 65 by cutting blood veins, 45 by jumping, and 35 by hanging (12), showing the differences in the methods of suicide in one KL and in one ghetto where more options were available.

The most frequent method to commit suicide in KL was hanging. In the early months of the Third Reich, camp guards often encouraged prisoners to kill themselves, even bringing them rope with which to do it. Suicide by hanging took place in isolated places, committed during night hours, when vigilance was lower, and there were many objects with which the inmates could commit suicide by hanging, such as belts, scarfs, or others, so giving prisoners rope with which to hang themselves was an act of mental torture.

Suicide through poisoning was very rare and used by prisoners who were members of the camp resistance movement and who had access to poisons or chemical substances. Some prisoners committed suicide by different poisons: iodine, cyanide, arsenic, strychnine, or even by swallowing cement (36). Other prisoners deliberately ventured across the SS guard lines to get shot. This method is reported in most of the camps (36, 57). Different authors have also reported that the contact with electrified barbed wire fences surrounding the camp was the most frequent form of suicide (58). However, these inmates killed on the fences or shot by guards would be classified as “killed in an escape attempt” instead of suicide.

Some authors (43, 58, 59) argue that some prisoners had chosen forms of suicide, which were not recognized as suicide, like “muslims.” The typical behavior of the so-called Muselmann (pl. *Muselmänner*) has been interpreted as a consequence of starvation but can be a bio–psycho–social process with an organic brain syndrome with apathy and affective disorders (60–62). There have been suggestions that “muslims” were inmates who have given up the will to live and thus could be regarded as concealed or passive suicides.

There are also descriptions of cases of mass suicide; it is reported that some soviet prisoners flung themselves onto the electric wires when they did not receive any food and water for days (27).

In most of cases, medical doctors in the camps falsified the primary cause of death, including suicide, on the prisoner’s death certificate. Sometimes, suicides were photographed by staff in various camps, as Auschwitz and Dachau (2, 59), but the meaning of this procedure is unknown.

### Aspects Related to Motivation and Suicidal Behavior

In Nazi KL, the desire to die in prisoners who committed suicide was deep, and they did not treat suicide as an act of demonstration as they did not want to gain the attention of others (63). Suicide was perceived by some as the last way of escape from unbearable conditions. For some prisoners, suicide was an opportunity to exercise free will and control, and the option of suicide was perceived as a human act of self-assertion (64). During the war phase of the existence of KL, the proximity of death erased borders between life and death to such an extent that it was not necessary to commit suicide (39).

Some authors (65, 66) distinguish three phases in the reactions of KL prisoners (a) initial shock with acute depersonalization; (b) complete exhaustion; and (c) despair just before the camp arrival. Affective life was reduced to a minimum, and the individual’s interests were limited to their immediate and most physical needs. The second phase is the adaptation as apathy state, as a self-protecting mechanism. There was also a pronounced irritability from a chronic lack of sleep and apathy because the prisoners were suffering from malnutrition. The third phase consists in a kind of depersonalization, regressive behavior, denial, isolation of affect, and discharge of aggression through alternative channels such as dreams (58, 61, 67).

We can differentiate two forms of behaviors when committing suicide in Nazi KL (a) impulsive behavior, such as crossing SS guard lines to get shot or touching the electrified barbed wire fences and (b) premeditated suicide, by hanging up or poisoning. These methods require more reflexion, looking for isolated places or poison to have. In case that we would consider self-starvation (“muslims”) as a suicide method, we could include it in this point.

Finally, it is noteworthy that some protective factors have been described (5, 68), such as desire to survive, familial responsibilities, children, fear of suicide or social censure, moral, or religious values. In Nazi KL, individual annulation and depersonalization eliminated most of these protective factors. Other factors such familial separation, suspicion of death of relatives, physical suffering, illness, hopelessness or extermination certainty extermination could eliminate capacity to survive (69).

## Soviet Special Camps

In May 1945, the Soviet secret service, the NKVD (*Naródniy komissariat vnútrennij del*), created special camps (70) in former KL located in Soviet-occupied territories. These camps did not have the same function as in the Nazi period; they were neither labor nor extermination camps. Nevertheless, living conditions were harsh and inmates were completely isolated from the outside world (71). In these camps, there was hunger and cold, most of the barracks were overfilled, and insufficient hygiene, sanitation, and nutrition lead to illness and epidemics (72). Nazi functionaries, including those responsible for block and cell units, members of SS, and Gestapo, and political prisoners sentenced by Soviet Military Tribunal were held in the camps, and others civilians were sent to these special camps without trial (73).

Data on suicides in Soviet special camps in Germany have not been published in the scientific literature, except the preliminary data provided by our group about Soviet Special Camp number 7, created in Sachsenhausen KL (74). The number of reported suicides in this camp under Soviet rule (1945–1950) was not significantly higher than in the general population (75) and much lower than the number reported when the camp was under Nazi rule. We calculated 2.8/10,000 suicides per year for the period from 1945 to 1950 against 11/10,000 suicides per year in the Nazi KL (74). This could be due to less atrocious conditions for prisoners, even when during the 5 years, 12,000 prisoners died from disease, hunger, and malnutrition. This can be explained by at least two reasons: first, it could be that not all suicides were reported as such by soviet camp authorities and second, it is reported that people with a major depressive disorder (and this could be the case of many German prisoners) do not have the motivation and energy required to commit suicide (14). In addition to this, finding the tools and opportunities to actively commit suicide in the Special Camps might not have been easy.

However, we want to emphasize that a high number of suicides committed by general population, Nazi leaders, and lower officials, occurred in Germany around the period of German surrender in 1945. During 1945, in the months around the end of the war, direct propaganda to the population exhorting to self-sacrifice and carrying cyanide capsules was quite common. Suicide levels reached their maximum in Berlin in April 1945 when no fewer than 3,881 people killed themselves (76).

The most commonly reported method for suicide in this Special Camp was hanging. Although it is not easy to establish, among the possible motivations for committing suicide, we can mention feelings of guilt for crimes committed, resignation or fear of punishment, and misguided understanding of the honor on the eve of criminal trials. Some authors reported suicidal tendencies in Nazi leaders (77), explaining that suicidal impulses were a result of the capture, trial, and condemnatory sentence (78), or alternatively, the Nazi regime’s brutality led to homicidal tendencies and turned into self-destructive behavior under the conditions of detention.

## Soviet Gulag Camps

The gulag (*Glavnoie Upravlenie LAGerei*) was a Soviet system of concentration camps established just after the Russian Revolution that lasted into the early 1980s, with a period of maximum activity between the late 1930s and the early 1950s. According to Applebaum (16), between 1929 and 1953, roughly 18 million Soviet citizens passed through the Gulag camps. But, if other people are also considered, such as exiled and prisoners of war, the total number could be up to almost 29 million. Soviet gulag camps differed from the Special camps created in Germany not only in the kind of prisoners (mainly political opponents) but also in the enforcement of the penalty of hard labor in order to support the industrialization of the Soviet Union (72).

Suicides in Soviet gulag camps have also been studied (29), although data on suicide rates in these camps are often widely conflicting. Mortality in Soviet gulag camps and labor colonies was 24.9% in 1942, 5.95% in 1945, and 0.95% in 1950 (29). It should be taken into account that infectious diseases, malnutrition, and hunger were the global challenges faced in the immediate postwar era. There are no official statistics available regarding the number of prisoners who attempted or completed suicide in gulags and some authors claim that suicides and mental illnesses were very rare (79, 80), while others report numerous accounts of suicide (16).

Maybe only active suicides were reported as such, but passive suicides were not. Self-destructive behaviors, such as self-mutilation in gulag camps or prisoners who let themselves die, have been widely reported. Self-mutilation was, in many cases, an attempt to save one’s life by escaping slave labor, being sent to the hospital, or even released as invalid (16). On the other hand, as happened in Nazi KL, those groups of dying prisoners suffering from infectious diseases, starvation, and vitamin deficiency were called *dokbodyagi* by gulag inmates (81). Some authors have described this behavior as a form of passive suicide (27).

## Conclusion

Suicides in KL are difficult to study because few documents are disposable, except data from interviews and testimonies. Moreover, there are a huge number of potential confounders in this topic: bias, because it is a self-report (recall bias), different religious, political and moral values, the setting (monitoring of the inmates, the kind and number of available tools), the time of imprisonment, etc. The topic of suicides in the Nazi KL has been studied more widely than in Soviet camps (Special camps and gulags), and there are marked differences between them, not only in the incidence rates (lower in the Soviet ones) but also in the possible motivation and suicidal behavior of suicidal inmates. The incidence of suicide in Nazi KL can be up to 10–30 times higher than for the general population. The main conclusions of topic analyzed are there are no specific profiles of suicidal group in the camps; the most frequent method to commit suicide was hanging; and the highest incidence of suicides occurs in the first years of imprisonment. Data on suicides during the Holocaust need to be analyzed in their fullness.

## Author Contributions

FL-M and EC-G both contributed ideas to the writing of the present mini-review.

## Conflict of Interest Statement

The authors declare that the research was conducted in the absence of any commercial or financial relationships that could be construed as a potential conflict of interest.
